# Negative Capability-Related Dimensions Among Local Government Public Health Nurses in Japan and Their Associated Factors

**DOI:** 10.7759/cureus.101208

**Published:** 2026-01-10

**Authors:** Masana Ujihara, Toru Onashi, Akane Suzuki, Masumi Watanabe, Koichi Ide, Tomoya Kawase, Teppei Masuya, Taeko Watanabe

**Affiliations:** 1 Faculty of Nursing and Nutrition, Shukutoku University, Chiba, JPN; 2 Faculty of Nursing, Iwate Prefectural University, Iwate, JPN; 3 Health Division, Miyako Public Health Center, Iwate, JPN; 4 Graduate School of Nursing, Shukutoku University, Chiba, JPN

**Keywords:** ambiguity tolerance, attitudes toward ambiguity, career development, japan, negative capability, public health nurses, resilience

## Abstract

Objective

Local government public health nurses (PHNs) in Japan deliver community-based public health services and often make practice decisions under uncertainty, incomplete information, and competing priorities. This study examined negative capability (NC)-related dimensions among PHNs in Japan - operationalized as ambiguity intolerance (MAT) and attitudes toward ambiguity - and explored factors associated with these dimensions.

Methods

We conducted an anonymous web-based survey of local government PHNs in Japan from July 16 to August 25, 2024. Ambiguity intolerance (indexed by higher MAT scores) was assessed using the Japanese 24-item Measure of Ambiguity Tolerance (MAT), and attitudes toward ambiguity were measured using a 26-item scale with five subscales (enjoyment, anxiety, reception, control, exclusion). Self-rated understanding of NC was assessed using a visual analogue scale (VAS, 0-100), resilience was measured using the 14-item Resilience Scale (RS14), and career development was measured using a 26-item career development scale for local government PHNs. Multiple linear regression (forced entry) was conducted for MAT and each attitude subscale, including sex, municipality type, years of PHN, RN, and other work experience, resilience, NC understanding, and career development subscales as explanatory variables. The primary outcome was ambiguity intolerance (MAT total score). The five attitude subscales (enjoyment, anxiety, reception, control, and exclusion) were treated as secondary exploratory outcomes.

Results

Data from 380 PHNs were analyzed (94.7% female; mean age 41.2 years, SD 11.9). The model for ambiguity enjoyment was significant (F(12,367)=9.03, p<0.001; adjusted R²=0.203) and showed positive associations with resilience (β=0.257, t=4.00, p<0.001), organizational mission/interpersonal support (β=0.168, t=2.27, p=0.024), professional identity as a PHN (β=0.147, t=2.29, p=0.023), and years of other work experience (β=0.103, t=2.20, p=0.029); years of PHN experience was not significantly associated (β=-0.122, t=-1.94, p=0.053). The model for ambiguity anxiety was significant (F(12,367)=9.82, p<0.001; adjusted R²=0.218) and showed negative associations with resilience (β=-0.357, t=-5.61, p<0.001), policy/organizational management competence (β=-0.270, t=-3.40, p<0.001), and years of PHN experience (β=-0.174, t=-2.79, p=0.006). Other attitude subscales (reception, control, and exclusion) also showed significant associations with several predictors, although effect sizes were modest. Of 532 submitted responses, 152 were excluded due to incomplete key variables. Adjusted R² ranged from 0.076 to 0.218 across outcomes, indicating modest explanatory power.

Conclusion

NC-related dimensions among local government PHNs were associated with both individual resources (resilience) and career development. Because the design was cross-sectional and outcomes captured NC-adjacent dimensions rather than NC itself, causal interpretation should be avoided; longitudinal, intervention, and qualitative studies are warranted. Because multiple outcomes and subscales were examined, there is an increased risk of chance findings; results should be interpreted cautiously and as hypothesis-generating.

## Introduction

Public health nurses (PHNs) employed by local governments in Japan engage in a wide range of practices spanning individual-level support and community-level implementation, including community assessment, planning, coordination with multiple stakeholders, and mobilization of social resources [1-3]. In routine practice and during large-scale crises, PHNs often face situations in which information is incomplete, priorities differ across stakeholders, and decisions must be made while continuously revising assessments over time [4]. Such practice requires not only technical knowledge but also the capacity to remain engaged with uncertainty and to proceed without relying on prematurely fixed conclusions [5-8].

Negative capability (NC) has been described as a capacity to stay with uncertainty and ambiguity without rushing to premature conclusions [5-8]. In helping professions, this orientation may support sustained engagement with complex situations that do not readily yield clear solutions [6-7]. From the perspective of public health nursing, long-term accompaniment of families facing intertwined social and health problems illustrates how practice may necessarily unfold within ambiguity and evolving relationships [9]. However, NC is a broad construct, and in quantitative research it is often approached through adjacent, more readily measurable constructs rather than a single, universally established instrument.

Given this measurement challenge, the present study focused on NC-related dimensions rather than NC itself. Specifically, we operationalized NC-related dimensions using ambiguity intolerance (MAT) and attitudes toward ambiguity [10-13]. Ambiguity intolerance reflects the extent to which ambiguous situations are experienced as threatening or uncomfortable, whereas attitudes toward ambiguity capture multiple facets such as positive engagement and anxiety [10-13]. Examining these dimensions may provide an empirically tractable approach to characterizing how PHNs relate to uncertainty in practice.

In addition, NC-related dimensions are likely to be shaped by both individual resources and career development. Resilience is commonly conceptualized as adaptive functioning under adversity [14], and has been measured in diverse populations using established instruments [15-16]. Career development in PHNs includes competencies and orientations that evolve through practice, such as organizational mission/interpersonal support, professional identity, and policy/organizational management [17]. Clarifying how these factors are associated with NC-related dimensions may inform the design of educational and organizational supports that help PHNs sustain practice in uncertain and demanding contexts.

Conceptually, we positioned resilience as an individual resource that may support adaptive engagement with uncertainty, and career development domains as practice-based competencies and orientations that may co-occur with NC-related dimensions. Although these constructs are assessed via self-report and are therefore subjective, they represent how practitioners appraise and respond to ambiguity, which is directly relevant to decision-making under uncertainty.

Therefore, this study aimed to describe NC-related dimensions among Japanese local government PHNs and to explore factors associated with these dimensions, focusing on ambiguity intolerance and attitudes toward ambiguity as outcomes.

## Materials and methods

Study design

This was a cross-sectional, web-based survey study.

Participants and procedure

This study targeted public health nurses (PHNs) employed by local governments in Japan (prefectures and municipalities). No additional inclusion or exclusion criteria were set beyond current employment as a local government PHN. Data were collected using an online questionnaire administered via SurveyMonkey (SurveyMonkey Inc., San Mateo, CA, USA).

Recruitment was conducted by distributing a study invitation to local government PHN leaders and relevant administrative contacts, who were asked to disseminate the survey link to eligible PHNs within their organizations. The invitation described the purpose of the study, the voluntary nature of participation, and the anonymity of responses. The survey was open from July 16, 2024, to August 25, 2024.

Sample size was estimated using a commonly used rule of thumb for multiple linear regression (N ≥ 50 + 8m, where m is the number of predictors) [18]. With m = 12, the minimum recommended sample size was 146. To obtain more stable estimates given the number of predictors, we set a target sample size of at least 300 participants.

Survey invitations were distributed to 500 municipal governments and 100 public health centers (PHCs) across Japan, requesting dissemination to local government public health nurses (PHNs). A total of 532 responses were submitted. We excluded 152 responses due to incomplete data on key study variables, leaving 380 PHNs for the analytic sample. For the regression analyses, missing data were handled using complete-case analysis within each model. Because dissemination was indirect, the response rate could not be determined.

Measures

Because a universally established instrument that directly measures the full construct of negative capability is not available, we assessed NC-related dimensions using (1) ambiguity intolerance (MAT) and (2) attitudes toward ambiguity.

Ambiguity intolerance was assessed using the Japanese 24-item Measure of Ambiguity Tolerance (MAT) developed and validated by Masuda, based on Norton’s MAT [10-11]. Items are rated on a 5-point Likert scale. Higher MAT scores indicate greater ambiguity intolerance (i.e., lower ambiguity tolerance), meaning that ambiguous situations are more likely to be experienced as threatening or uncomfortable [11]. In the present study, the total score was used in the analyses.

Attitudes toward ambiguity were measured using Nishimura’s Attitudes towards Ambiguity Scale, which conceptualizes attitudes as a multi-dimensional structure [12]. The scale comprises five subscales - enjoyment, anxiety, reception, control, and exclusion - rated on a Likert-type response format [12]. For each subscale, higher scores indicate a stronger tendency toward the corresponding attitude. Subscale scores were used as dependent variables in the regression analyses.

Resilience was assessed with the 14-item Resilience Scale (RS-14) [15-16]. Items are rated on a Likert scale, and higher total scores indicate higher resilience [15]. The total score was used in the analyses. The RS-14 is copyrighted by Gail M. Wagnild, PhD, RN, and was used with permission.

In this study, resilience was conceptualized as an individual resource potentially associated with NC-related dimensions, rather than as an NC measure.

Career development was assessed using a career development scale for public health nurses, which consists of five domains: policy and organizational management, organizational mission and interpersonal support, community health activities, job and workplace satisfaction, and PHN professional consciousness [17]. Domain scores were used as independent variables in the regression analyses.

Self-rated understanding of negative capability was assessed using a visual analog scale (VAS). Participants rated how well they understood the concept of NC on a 0-100 scale, with higher scores indicating greater self-rated understanding. Because this measure reflects perceived understanding, it does not necessarily represent objective knowledge of NC.

All psychometric instruments used in this study have been previously published and are cited in the text (MAT; Attitudes toward Ambiguity Scale; RS-14; PHN career development scale). Scale scoring followed the original publications. Where required, permission to use the instruments was obtained from the copyright holders.

Statistical analysis

Descriptive statistics were calculated for participant characteristics and study variables. Continuous variables are presented as means and standard deviations (SD), and categorical variables as counts and percentages.

To explore factors associated with NC-related dimensions, we conducted multiple linear regression analyses using the forced-entry method. Dependent variables were (1) the total score of ambiguity intolerance (MAT) and (2) each subscale score of the attitudes toward ambiguity scale (enjoyment, anxiety, reception, control, and exclusion). Independent variables were sex, type of municipality, years of experience as a public health nurse, years of experience as a registered nurse (RN), years of other work experience, resilience (RS-14 total score), five domains of career development (career development scale), and self-rated understanding of negative capability (VAS score).

The primary outcome was ambiguity intolerance measured by the MAT total score. The five attitude subscales (enjoyment, anxiety, reception, control, and exclusion) were analyzed as secondary exploratory outcomes. Parametric methods were used because the outcomes were total/subscale scores derived from multiple items and have been treated as approximately continuous in prior validation studies; additionally, the sample size was sufficiently large for linear-model inference to be robust to modest departures from normality. We used forced-entry multiple linear regression to estimate associations between the outcomes and candidate predictors defined a priori based on theoretical and prior empirical considerations, rather than data-driven selection. Beyond multicollinearity (VIF), we did not formally evaluate all regression assumptions (e.g., linearity, residual normality, and homoscedasticity); thus, some model misspecification cannot be ruled out. Because we examined multiple outcomes and subscales, there is an increased risk of type I error. We did not apply formal multiplicity corrections because the analyses were exploratory and intended to generate hypotheses; therefore, findings should be interpreted cautiously, with emphasis on effect sizes and consistency rather than p-values alone.

Sex and municipality type were entered as dummy variables. For sex, male was used as the reference category; for municipality type, municipal governments/cities with a public health center were used as the reference category. Standardized regression coefficients (β), t statistics, and two-sided p-values are reported for predictors; overall model fit is summarized using the F statistic and adjusted R-squared.

Multicollinearity was assessed using variance inflation factors (VIFs). Statistical significance was set at p < 0.05 (two-tailed). All analyses were performed using IBM SPSS Statistics for Windows, Version 29.0 (IBM Corp., Armonk, NY, USA).

Ethics and consent statements

Ethics Statement

This study was approved by the Shukutoku University Research Ethics Review Committee (approval number: N24-01).

Consent Statement

On the first page of the survey, participants were informed about the study purpose, voluntary participation, anonymity, and publication of aggregated findings. Completion of the questionnaire was regarded as informed consent.

## Results

Participant characteristics and scale scores

A total of 380 local government public health nurses (PHNs) were included in the analyses (Table 1). Most participants were women (n = 360, 94.7%). The mean age was 41.2 years. Regarding educational background, junior college or university advanced courses were the most common (n = 242, 63.7%), followed by vocational school (n = 103, 27.1%) and master’s degree programs (n = 27, 7.1%).

**Table 1:** Participant characteristics of local government public health nurses Note: N = 380. ^a^ Mean; ^b^ SD. Age is presented as mean (SD). ^c^ Years of other work experience exclude nursing and PHN experience. PHN: Public health nurse; RN: Registered nurse

Variable	Category	n	%
Sex	Female	360	95
	Male	20	5
Age (years)		41.2^a^	11.9^b^
Type of municipality	Municipal government	224	58.9
	Prefectural government	112	29.5
	City with a public health center	44	11.6
Workplace	Public health center	127	33.4
	City/town/village office	126	33.2
	Community health center	104	27.4
	Comprehensive Support Center for Families with Children	10	2.6
	Community Comprehensive Support Center	8	2.1
	Other	5	1.3
Current work area(s)	Maternal and child health	101	26.6
	Adult health	84	22.1
	Infectious disease control	42	11.1
	Older adult health / long-term care prevention	29	7.6
	Mental health	25	6.6
	Intractable disease control	14	3.7
	Child abuse prevention	12	3.2
	National Health Insurance Database system-related	8	2.1
	Disability health	3	0.8
	Suicide prevention	2	0.5
	Occupational health	1	0.3
	Other	59	15.5
Years of PHN experience	0-2	59	15.5
	3-5	53	13.9
	6-10	61	16.1
	11-19	70	18.4
	20-	137	36.1
Years of RN experience	0-5	342	90
	6-	38	10
Years of other work experience^c^	0-5	371	97.6
	6-	9	2.4
Highest education	Junior college/university advanced course	242	63.7
	Vocational school	103	27.1
	Graduate school (Master’s)	27	7.1
	Other	8	2.1

Descriptive statistics for NC-related dimensions (ambiguity intolerance [MAT] and attitudes toward ambiguity: enjoyment, anxiety, reception, control, and exclusion), resilience (RS-14), career development domains, and self-rated understanding of negative capability are presented in Table 2. The mean RS-14 score was 63.86 (SD = 12.20).

**Table 2:** Descriptive statistics of scale scores Note: N = 380. MAT, Measure of Ambiguity Tolerance [10-11] (higher scores indicate greater ambiguity intolerance / lower ambiguity tolerance). Attitudes toward Ambiguity Scale (enjoyment, anxiety, reception, control, exclusion) [12] (higher scores indicate stronger endorsement of each attitude). RS-14, 14-item Resilience Scale [15-16] (higher scores indicate higher resilience). Career development scale for local government PHNs [17]. VAS ranges from 0 to 100 (higher scores indicate greater self-rated understanding of NC).

Variable	Mean	SD
Ambiguity intolerance (MAT)	67.2	13.1
Attitudes toward ambiguity		
Ambiguity enjoyment	29.13	5.71
Ambiguity anxiety	21.28	5.6
Ambiguity reception	19.59	4.61
Ambiguity control	21.3	4.22
Ambiguity exclusion	9.17	3.06
Resilience	63.86	12.2
Career development		
Organizational mission and interpersonal support	21.92	3.19
Community health activities	12.88	2.95
Policy and organizational management	17.97	5.02
PHN professional consciousness	17.44	4.29
Job and workplace satisfaction	17.47	3.61
Self-rated understanding of NC	27.03	26.41

Multiple regression analyses

Results of the multiple linear regression analyses are shown in Table 3. Model explanatory power varied across dependent variables, with adjusted R-squared values indicating modest fit overall (adjusted R² = 0.076-0.218). Multicollinearity was not considered problematic; variance inflation factors (VIFs) ranged from 1.037 to 3.048.

**Table 3:** Multiple linear regression analyses with ambiguity intolerance (MAT) and attitudes toward ambiguity as dependent variables Note: MAT, Measure of Ambiguity Tolerance [10-11] (higher scores indicate greater ambiguity intolerance); Attitudes toward Ambiguity Scale (enjoyment, anxiety, reception, control, exclusion) [12]; RS-14, 14-item Resilience Scale [15-16]; Career development scale for local government PHNs [17]. Values are standardized β (t statistics and p-values in parentheses). Dependent variables are Ambiguity Intolerance (MAT) total score and attitudes toward ambiguity subscales. Municipality type: 0=municipal government/city with PHC, 1=prefectural government. VIF: variance inflation factor.

Explanatory variables	Ambiguity intolerance (MAT)	Ambiguity enjoyment	Ambiguity anxiety	Ambiguity reception	Ambiguity control	Ambiguity exclusion
Sex (0=male, 1=female)	0.009 (0.19, 0.850)	0.038 (0.80, 0.425)	0.033 (0.71, 0.477)	0.002 (0.04, 0.968)	−0.014 (−0.28, 0.779)	0.016 (0.32, 0.752)
Municipality type (0=municipal/city with PHC, 1=prefecture)	0.022 (0.45, 0.650)	−0.002 (−0.05, 0.962)	−0.008 (−0.16, 0.869)	−0.022 (−0.45, 0.655)	−0.007 (−0.13, 0.895)	0.020 (0.40, 0.689)
Years of PHN experience	0.190 (2.89, 0.004)	−0.122 (−1.94, 0.053)	−0.174 (−2.79, 0.006)	0.212 (3.14, 0.002)	−0.298 (−4.42, < 0.001)	−0.219 (−3.24, 0.001)
Years of RN experience	0.117 (2.34, 0.020)	−0.004 (−0.09, 0.928)	−0.082 (−1.73, 0.084)	0.052 (1.02, 0.308)	−0.048 (−0.94, 0.348)	−0.078 (−1.51, 0.131)
Years of other work experience	0.021 (0.44, 0.664)	0.103 (2.20, 0.029)	−0.062 (−1.33, 0.184)	0.039 (0.78, 0.438)	−0.011 (−0.22, 0.829)	−0.051 (−1.01, 0.314)
RS-14 total	0.262 (3.88, < 0.001)	0.257 (4.00, < 0.001)	−0.357 (−5.61, < 0.001)	0.174 (2.52, 0.012)	−0.124 (−1.80, 0.072)	−0.147 (−2.12, 0.035)
Career development						
Organizational mission and interpersonal support	−0.140 (−1.80, 0.072)	0.168 (2.27, 0.024)	0.131 (1.78, 0.075)	−0.005 (−0.06, 0.952)	0.238 (3.00, 0.003)	0.230 (2.89, 0.004)
Community health activities	0.011 (0.14, 0.885)	0.024 (0.32, 0.747)	0.051 (0.70, 0.483)	−0.079 (−1.01, 0.312)	0.086 (1.10, 0.271)	0.014 (0.18, 0.857)
Policy and organizational management	0.104 (1.23, 0.218)	0.004 (0.05, 0.961)	−0.270 (−3.40, < 0.001)	0.015 (0.17, 0.863)	−0.074 (−0.86, 0.388)	−0.087 (−1.01, 0.311)
Professional identity as a PHN	0.046 (0.68, 0.495)	0.147 (2.29, 0.023)	0.088 (1.39, 0.165)	−0.133 (−1.93, 0.054)	0.084 (1.23, 0.221)	−0.031 (−0.44, 0.658)
Job/workplace satisfaction	0.034 (0.51, 0.608)	−0.075 (−1.18, 0.239)	0.033 (0.52, 0.604)	0.153 (2.25, 0.025)	−0.032 (−0.47, 0.636)	−0.150 (−2.20, 0.028)
Self-rated understanding of NC	−0.047 (−0.89, 0.375)	0.087 (1.72, 0.086)	−0.024 (−0.48, 0.631)	0.087 (1.61, 0.107)	0.102 (1.88, 0.061)	0.060 (1.11, 0.268)
Adjusted R²	0.122	0.203	0.218	0.08	0.082	0.076
Model F (df1, df2), p	F(12,367)=5.37, p< 0.001	F(12,367)=9.03, p< 0.001	F(12,367)=9.82, p< 0.001	F(12,367)=3.73, p< 0.001	F(12,367)=3.81, p< 0.001	F(12,367)=3.60, p< 0.001
VIF (min–max)	1.037–3.048					

For ambiguity enjoyment, the overall model was statistically significant (F(12, 367) = 9.03, p < 0.001; adjusted R² = 0.203). Higher resilience (RS-14) was associated with higher enjoyment (β = 0.257, t = 4.00, p < 0.001). Ambiguity enjoyment was also positively associated with organizational mission and interpersonal support (β = 0.168, t = 2.27, p = 0.024), professional identity as a PHN (β = 0.147, t = 2.29, p = 0.023), and years of other work experience (β = 0.103, t = 2.20, p = 0.029). Years of PHN experience was not significantly associated with enjoyment (β = −0.122, t = −1.94, p = 0.053).

For ambiguity anxiety, the overall model was statistically significant (F(12, 367) = 9.82, p < 0.001; adjusted R² = 0.218). Higher resilience (RS-14) was associated with lower anxiety (β = −0.357, t = −5.61, p < 0.001). Stronger policy and organizational management competence (β = −0.270, t = −3.40, p < 0.001) and longer PHN experience (β = −0.174, t = −2.79, p = 0.006) were also associated with lower ambiguity anxiety.

For the remaining outcomes (ambiguity intolerance [MAT total], reception, control, and exclusion), explanatory power was smaller (adjusted R² = 0.076-0.122), although several predictors showed statistically significant associations in some models.

## Discussion

Principal findings

This study examined NC-related dimensions among Japanese local government public health nurses (PHNs), operationalized as ambiguity intolerance (MAT) and attitudes toward ambiguity, and explored associated factors. PHNs in Japan are expected to provide individual-level support while coordinating and implementing community-level public health programs within local government systems [1-3, 19]. In this context, practice often requires working under incomplete information and value conflicts, and repeatedly updating assessments while coordinating stakeholders [9]. Our findings suggest that NC-related dimensions were associated with both individual resources (resilience) and aspects of career development.

Specifically, higher ambiguity enjoyment was associated with higher resilience, higher scores in career development domains reflecting organizational mission/interpersonal support and professional identity, and longer other work experience. Lower ambiguity anxiety was associated with higher resilience, stronger policy/organizational management, and longer PHN experience. Other outcomes (ambiguity intolerance, reception, control, and exclusion) showed smaller or non-significant associations in the fitted models.

Interpretation of associations

Resilience showed consistent associations with both higher ambiguity enjoyment and lower ambiguity anxiety. Resilience is commonly conceptualized as adaptive functioning under adversity [14], and has been examined among nurses and other health professionals in relation to work-related stress and coping [20-23]. In our data, resilience may reflect a broad capacity to maintain functioning under pressure that co-occurs with more adaptive attitudes toward ambiguity. However, because the design is cross-sectional, reverse direction and mutual reinforcement are also possible; for example, a stance that allows one to remain engaged in ambiguous situations may be reflected in higher resilience scores over time.

Career development domains were also associated with attitudes toward ambiguity. Ambiguity enjoyment was associated with organizational mission/interpersonal support and professional identity. In PHN practice, professional identity and the meaning attributed to one’s role have been discussed as important in sustaining practice and motivation [24]. These orientations may be linked to how PHNs approach uncertainty in practice, including whether they continue information gathering and coordination rather than disengaging when solutions are not immediately clear. Similarly, lower ambiguity anxiety was associated with policy/organizational management. This domain may reflect competencies related to organizing resources and procedures and coordinating within organizations, which could be relevant in situations where uncertainty cannot be eliminated but must be managed through structured decision-making and coordination.

Years of PHN experience was negatively associated with ambiguity anxiety. This may reflect repeated exposure to uncertain situations and accumulated experiential learning. At the same time, selection processes are plausible; for example, PHNs with lower anxiety in ambiguous settings may be more likely to remain in roles that involve coordination and management. Therefore, these associations should not be interpreted as evidence that experience or organizational management competencies directly reduce anxiety.

Other work experience was positively associated with ambiguity enjoyment. Prior work experience outside PHN roles may provide opportunities to encounter diverse organizational cultures and decision-making styles, potentially broadening one’s repertoire for handling uncertain situations. This interpretation remains tentative and requires further investigation, including qualitative exploration of what types of experience are most relevant.

Practical implications

Our results highlight that NC-related dimensions among PHNs are associated with both individual resources and career development. From an implementation perspective, supports that combine individual-level learning with organizational and supervisory processes may be relevant. In PHN education and training, structured reflection on decision-making under uncertainty, case-based discussion, and supervision that makes implicit reasoning explicit may help PHNs develop ways to proceed while updating assessments rather than seeking premature closure. In addition, training that strengthens competencies related to policy/organizational management and coordination may be relevant for managing uncertainty in system-level responses, as described in reports of PHN management roles during the early COVID-19 period [4, 25-27]. Because this study does not establish causal relationships, these implications should be considered as hypotheses for future program development and evaluation rather than as definitive intervention recommendations. As noted above, the present analyses address NC-related dimensions (ambiguity intolerance and attitudes toward ambiguity) rather than NC itself, and the models explained a modest proportion of variance (adjusted R² = 0.076-0.218). Given the exploratory structure with multiple outcomes, these implications should be viewed as hypotheses that warrant replication and evaluation.

Limitations and future directions

Several limitations should be noted. First, this was a web-based survey, and selection bias is possible. The number of PHNs reached through dissemination and the response rate could not be determined. Second, we did not measure NC directly; instead, we assessed NC-related dimensions using ambiguity intolerance (MAT) and attitudes toward ambiguity [10-12]. Higher MAT total scores indicate lower ambiguity tolerance (i.e., greater ambiguity intolerance), which should be considered when interpreting findings. Third, self-rated understanding of NC was measured by a VAS and reflects perceived understanding rather than objective knowledge. Fourth, as a cross-sectional study, temporal ordering cannot be established, and unmeasured confounding is likely (e.g., workload, organizational support, leadership roles, and local context). Fifth, all measures were self-reported at a single time point, which may have introduced reporting-related bias. Sixth, a substantial proportion of submitted responses were excluded due to missing key variables (152/532), and regression analyses used complete-case analysis within each model. If missingness was related to outcomes and/or predictors, estimates may be biased and external validity may be reduced. Seventh, because multiple outcomes and subscales were examined without multiplicity correction in this exploratory analysis, there is an increased risk of type I error, and findings should be interpreted cautiously. Finally, we did not formally evaluate all regression assumptions beyond multicollinearity; thus, residual model misspecification cannot be fully ruled out.

Future research should examine temporal ordering using longitudinal designs and evaluate specific training and organizational support strategies using intervention studies. Qualitative studies may clarify how PHNs maintain engagement with uncertainty in practice, how professional identity and organizational competencies shape decision-making processes, and what contextual mechanisms link career development to attitudes toward ambiguity. Further work is also needed to refine measurement approaches for NC-related constructs, and to examine their relationships with relevant outcomes such as burnout and well-being among health professionals [28]. Conceptual work connecting NC and adjacent constructs may also support clearer interpretation and hypothesis generation in PHN practice contexts [5-6, 29-30].

## Conclusions

In this cross-sectional web-based survey of 380 Japanese local government public health nurses, NC-related dimensions operationalized as ambiguity intolerance and attitudes toward ambiguity were associated with both individual resources (resilience) and aspects of career development. Higher ambiguity enjoyment was associated with higher resilience and career development domains reflecting organizational mission/interpersonal support and professional identity, whereas lower ambiguity anxiety was associated with higher resilience, stronger policy/organizational management, and longer PHN experience. These findings should be interpreted as associations involving NC-related dimensions rather than NC itself, and causal inferences cannot be made. Longitudinal, qualitative, and intervention studies are needed to clarify temporal ordering, contextual mechanisms, and the specific educational and organizational supports that may help PHNs sustain practice under uncertainty.
